# Complications of Estimating Hatchery Introgression in the Face of Rapid Divergence: A Case Study in Brook Trout (*Salvelinus fontinalis*)

**DOI:** 10.1111/eva.70026

**Published:** 2024-12-16

**Authors:** Bradley Erdman, Wesley Larson, Matthew G. Mitro, Joanna D. T. Griffin, David Rowe, Justin Haglund, Kirk Olson, Michael T. Kinnison

**Affiliations:** ^1^ School of Biology and Ecology, Ecology and Environmental Sciences Program University of Maine Orono Maine USA; ^2^ Department of Biological Sciences University of New Hampshire Durham New Hampshire USA; ^3^ Auke Bay Laboratories, Alaska Fisheries Science Center, National Marine Fisheries Service National Oceanic and Atmospheric Administration Juneau Alaska USA; ^4^ Wisconsin Department of Natural Resources Office of Applied Science Madison Wisconsin USA; ^5^ Wisconsin Department of Natural Resources Bureau of Watershed Management Madison Wisconsin USA; ^6^ Wisconsin Department of Natural Resources Bureau of Fisheries Management Fitchburg Wisconsin USA; ^7^ Wisconsin Department of Natural Resources Bureau of Fisheries Management Dodgeville Wisconsin USA; ^8^ Wisconsin Department of Natural Resources Bureau of Fisheries Management La Crosse Wisconsin USA; ^9^ Maine Center for Genetics in the Environment University of Maine Orono Maine USA

**Keywords:** brook trout, fisheries management, genomics, hatchery introgression, salmonid, simulation

## Abstract

Fish stocking has been utilized for over a century to offset extirpations or declines in abundance of many native species. These historical declines and hatchery contributions have led to uncertainty surrounding whether many contemporary populations are native, introgressed with hatchery sources, or entirely of hatchery origin. Such uncertainty is problematic for the conservation of native biodiversity as it hampers management agencies' ability to prioritize the conservation of indigenous locally adapted populations. Fortunately, genetic and genomic tools have allowed researchers to investigate these questions, often through the use of clustering or assignment approaches that are predicated on identifiable and consistent divergence between native populations and hatchery sources. Here, we apply these methods to restriction‐site associated DNA (RAD) data from 643 brook trout (*Salvelinus fontinalis*) originating from 13 wild populations and an exogenous hatchery strain to investigate the extent of historical extirpations, hatchery contributions, and processes affecting population structure in a small area of the previously unglaciated Driftless Area of Wisconsin, USA. The results from these analyses suggest that wild populations in this region are genetically distinct even at small spatial scales, lack strong hydrologically associated population structure, rarely exchange gene flow, and have small effective population sizes. Furthermore, wild populations are substantially diverged from known hatchery strains and show minimal evidence of introgression in clustering analyses. However, we demonstrate through empirically informed simulations that distinct wild populations may potentially be hatchery‐founded and have since diverged through rapid genetic drift. Collectively, the apparent lack of hydrological population structure and potential for rapid drift in the Driftless Area suggest that many native populations may have been historically extirpated and refounded by stocking events. If this is the case, then commonly used genomic clustering methods and their associated model selection criteria may result in underestimation of hatchery introgression in the face of rapid drift.

## 1 Introduction

Natural selection in response to environmental heterogeneity creates a mosaic of locally adapted populations that are integral for long term stability of metapopulations and species (Luck, Daily, and Ehrlich 2003; Kawecki and Ebert 2004; Schindler et al. 2010; Schindler, Armstrong, and Reed 2015). Accordingly, there has been an increasing focus on conserving indigenous populations and the local adaptations they contain (Allendorf et al. 2001; Garcia de Leaniz et al. 2007; Razgour et al. 2019). However, historical human‐mediated species dispersal and introductions has resulted in uncertainty regarding whether populations are of native, introduced, or introgressed origin, hindering the ability of conservation practitioners to prioritize the conservation of indigenous populations (e.g., Habera and Moore 2005). These uncertainties are perhaps most prevalent in economically important fishes, which have a long history of supplementation with hatchery‐derived conspecifics to offset anthropogenic declines in abundance (Trushenski, Whelan, and Bowker 2018). Indeed, stocking continues to be one of the most ubiquitous fisheries management strategies in North America and upwards of a billion fish are stocked into inland, coastal, and marine waters annually, and often into preexisting wild populations (Halverson 2008).

Advancements of genetic techniques and an increasing focus on preserving local adaptations have prompted numerous investigations of hatchery introgression for many fishes (e.g., Turnquist et al. 2017; Kazyak et al. 2018; Bootsma et al. 2021; Homola et al. 2021). The extent of hatchery introgression is variable across species, study systems, and their respective management histories (e.g., Araki and Schmid 2010; Turnquist et al. 2017; Kazyak et al. 2018; Lehnert et al. 2020; Bootsma et al. 2021; Homola et al. 2021). Although for some species such as brook trout (*Salvelinus fontinalis*), low proportions of hatchery introgression are predominantly observed (Kazyak et al. 2018; White et al. 2018; Beer et al. 2019; Lehnert et al. 2020) and these observations are often attributed to reduced fitness of exogenous or domesticated hatchery sources (Berejikian and Ford 2004; Araki, Cooper, and Blouin 2007; Araki et al. 2008; Christie, Ford, and Blouin 2014). Such claims are often supported by evidence that supplemented populations appear notably divergent from hatchery sources, and that this divergence can even increase with time since stocking cessation (Perrier et al. 2013; Harbicht, Wilson, and Fraser 2014; Valiquette et al. 2014; Létourneau et al. 2018; Erdman, Mitro, et al. 2022, Erdman, Gallagher, et al. 2022). This phenomenon is particularly relevant to the conservation of native biodiversity as it suggests that natural selection may purge maladaptive hatchery‐derived alleles over time. However, the hatchery introgression estimates underlying these trends are typically obtained by comparing contemporary wild and hatchery samples and increasing dissimilarity over time might also be explained, at least in part, by genetic drift. Resolving these two competing hypotheses (i.e., drift vs. purging of introgressed alleles) is important as they may significantly influence priorities for in situ conservation or which wild sources are selected for reintroduction programs. Yet, few studies strongly consider drift as an alternate explanation for the divergence of stocked populations and hatchery sources. This is potentially problematic given that many investigations of hatchery introgression focus on salmonid populations that often exhibit very small effective population sizes and have the potential to neutrally diverge from hatchery sources over relatively rapid timescales comparable to stocking histories (e.g., Kazyak et al. 2022). Furthermore, many studies utilize modern hatchery reference collections that may themselves have substantially diverged from historical hatchery strains and thus pose the risk of chronically underestimating historical introgression. This phenomenon is also applicable to discontinued, historical hatchery strains where no collections are available, and may pose the risk of misattributing divergent, but nonetheless hatchery founded, populations as remnant indigenous populations. Similarly, such rapid divergence can also apply to naturally recolonized populations and, without further investigation of demographic parameters (e.g., effective population size, gene flow), confound recently refounded and longstanding populations, with corresponding implications for the scope of local adaptation.

Brook trout are an economically important sportfish that occupy a diverse array of habitats and have faced declines in many parts of their native range (Hudy et al. 2008). These declines have been particularly pronounced in the unglaciated Driftless Area of Minnesota, Wisconsin, and Iowa, resulting from extensive land clearing, agricultural practices, and overfishing beginning in the 1850s (Trimble and Lund 1982; Thorn et al. 1997; Vetrano 2017). This time period coincided with the advent of modern fish culture and brook trout were soon stocked throughout the region to offset these declines (Wisconsin Fish Commission 1877; Thorn et al. 1997). Improved land‐use practices began to be implemented in the 1930s and throughout the following decades potentially remediated stream conditions to an extent that allowed for increased abundance and re‐establishment of extirpated populations (Trimble and Lund 1982; Thorn et al. 1997; Olson, Mauel, and Pechacek 2021; Olson 2023). Contemporary populations can now be found throughout the region and the sport fishery contributes hundreds of millions of dollars to local economies annually (Anderson 2016). Brook trout populations elsewhere in their native range are typified by genetic population structure that follows hierarchical hydrological boundaries (i.e., small streams to large watersheds, hereafter referred to hydrological population structure; Lehnert et al. 2020; Morgan et al. 2021; Erdman, Gallagher, et al. 2022; Kazyak et al. 2022). Populations that deviate from this pattern can often be explained by anthropogenic disturbances, such as habitat fragmentation or stocking. In the latter case, populations exhibit genetic similarities to hatchery strains while being differentiated from the hydrologically structured native populations elsewhere in the region. However, the genetic origins of contemporary populations in the Driftless Area remain disputed due to a lack of hydrological population structure and high levels of divergence between both wild populations and hatchery strains (Hughes 2008; Erdman, Mitro, et al. 2022). In this sense, the lack of hydrological population structure suggests a non‐native origin, but the concurrent genetic dissimilarity from hatchery strains suggests minimal hatchery introgression or influence.

In this study, we conduct the first genomic investigation of brook trout in the Driftless Area of Wisconsin to resolve population structure and quantify the extent of hatchery introgression in wild populations. While genetic studies have been conducted to investigate broadscale patterns of population structure throughout the Midwestern United States (Erdman, Mitro, et al. 2022), here we focus on a smaller spatial extent to investigate the demographic processes (i.e., gene flow and genetic drift) that might be expected to contribute to the maintenance of population structure. Many of these populations have small effective population sizes and exchange minimal natural gene flow, making this an apt case study to explore the potential for genetic drift to confound estimates of historical hatchery introgression. We accordingly use empirically informed simulations of genetic drift to ascertain our analytical power to quantify hatchery introgression in the face of rapid neutral divergence. Collectively, these results suggest a complex history of habitat degradation, hatchery contributions, processes affecting population structure (i.e., low gene flow and rapid genetic drift), and potential pitfalls of commonly used hatchery introgression estimation methods for populations outside of drift‐migration equilibria.

## 2 Methods

### 2.1 Stocking History

Wisconsin has a longstanding history of both private and State‐sponsored fish culture (Wisconsin State Conservation Commission 1930). The first brook trout stockings likely originated from private hatcheries in the late 1860s and there is evidence that some of these early operations were substantial, with a single brook trout hatchery recorded as producing 250,000 individuals in 1876 (Wisconsin Fish Commission 1876). Indeed, public interest of fish culture was robust with at least 81 Wisconsin residents interested in or actively practicing fish culture in 1882 (United States Fish Commission 1882). The State of Wisconsin eventually became involved in hatchery operations and began maintaining captive brook trout broodstock in 1876 after obtaining 2000 adults from a private hatchery near Waterville, Wisconsin (Wisconsin Fish Commission 1876). Progeny from these broodstock were first used to stock public waters in 1877 and by 1881 the State was stocking over a million fry per year (Wisconsin Fish Commission 1877, 1881). The State of Wisconsin transitioned to stocking the Osceola strain in 1925, and these fish are believed to have been sourced from the Rock Creek Hatchery (St. Croix Falls, Wisconsin; Hunt 1979). However, the nomenclature of the Osceola strain suggests the lineage likely originated from the larger Troutmere Hatchery (Osceola, Wisconsin) that sourced broodfish from Close's Creek on their property (Callen 1983; Fields and Philipp 1998; Hoverman B., Osceola Historical Society, pers. comm.). Thus, it appears that at least some of these early stockings utilized regional sources, but origins of many other private hatcheries' progeny remain unknown, and it is therefore plausible that exogenous sources were also imported from other states and provinces during this time (Wisconsin State Conservation Commission 1930; Krueger and Menzel 1979).

The Osceola strain was abandoned in 1973 and new hatchery strains were imported from facilities located along the Atlantic seaboard (Hunt 1979; Callen 1983; Fields and Philipp 1998). The most prevalent strain used since this time has been the nominal St. Croix Falls hatchery strain that was initially developed at the Paint Bank Fish Hatchery (Paint Bank, Virginia) before being transferred to the Nashua National Fish Hatchery (Nashua, New Hampshire) and eventually the St Croix Falls State Fish Hatchery in 1973 (St. Croix Falls, Wisconsin; Claggett and Dehring 1984; Fields and Philipp 1998; Hoxmeier, Dieterman, and Miller 2015). In addition, Randolph x Assinica F_1_ hybrids, representing parental strains of unknown wild and Assinica Lake (Québec, Canada) origin, respectively, were briefly stocked on an experimental basis (Moyle 1969; Flick and Webster 1976; Hunt 1979; Perkins, Krueger, and May 1993). Finally, the Wisconsin Department of Natural Resources (WDNR) has also utilized several locally derived strains since the 1990s and also conducted wild translocations from the late 1970s to 2007 (Wisconsin Department of Natural Resource 2019; Van Dyck G., WDNR, pers. comm.). Population‐specific stocking records are only available post‐1972 but indicate increasing brook trout stocking in the Driftless Area with approximately 86,000 fingerling (length = ~62 mm) or larger individuals stocked into an average of 99 streams per year in the 1970s, and increasing to approximately 171,000 individuals stocked into an average of 149 streams in the 2010s.

### 2.2 Sampling

**FIGURE 1:**
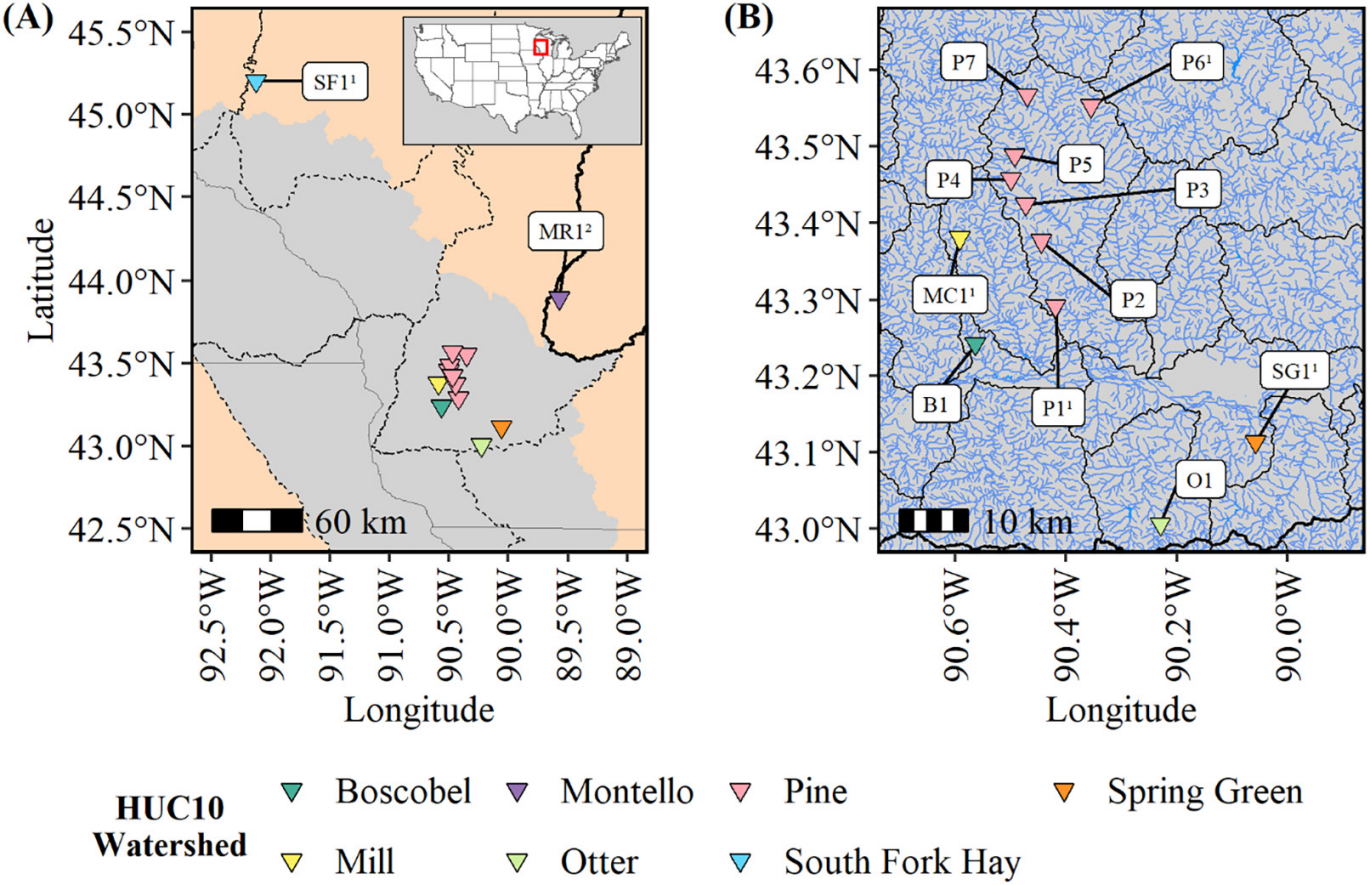
(A) Sampling locations of 13 wild brook trout populations color‐coded by HUC10 watershed. Thick black lines denote HUC2 watershed boundaries, thin dashed lines denote HUC4 watershed boundaries, and the grey area denotes the extent of the Driftless Area. Superscripts denote populations used as wild broodsources (^1^) or translocation sources (^2^). (B) Sampling locations of 11 wild brook trout populations in the Driftless Area color‐coded by HUC10 watershed. Thick black lines denote HUC4 watershed boundaries and thin dashed lines denote HUC10 watershed boundaries. Full population names can be matched with population‐specific watershed abbreviations found in Table 1.

Fin clips were collected from 13 wild brook trout populations in Wisconsin and the St. Croix Falls hatchery strain with a target sample size of 50 fish per population (Table 1 and Figure 1). All wild samples were collected via backpack electrofishing from 2015 to 2019, St. Croix Falls hatchery strain samples were collected from broodstock maintained at the St. Croix Falls State Fish Hatchery in 2019, and all fin clips were preserved in 95% ethanol prior to DNA extraction. Our primary objective for these collections was to examine putative fine scale population structure of populations originating from the Pine River watershed in the Driftless Area of Wisconsin. To this aim, we collected samples from seven populations in the Pine River watershed, of which two (Ash and Melancthon creeks) have also served as broodstock sources. Given the diverse stocking sources used in Wisconsin, we also included four additional populations outside of the Pine River watershed (West Branch of Mill Creek, Lowery Creek, South Fork of the Hay River, and Lawrence Creek) that have historically or currently serve as wild brood or translocation sources. In addition, we also sampled two additional populations near the Pine River watershed in the Driftless Area (Figure 1).

**TABLE 1:** Sample collection information and associated measures of genetic diversity for 13 wild brook trout populations and the St. Croix Falls Hatchery strain. Code is a population‐specific abbreviation where letters correspond to HUC10 watersheds (see Figure 1 for geographic locations). Genetic diversity metrics include allelic richness (*A*_*r*_), observed heterozygosity (*H*_*o*_), expected heterozygosity (*H*_*e*_), inbreeding coefficients (*F*_*is*_), effective population sizes (*N*_*e*_), and corresponding jackknifed 95% confidence intervals.

Population	Code	*N*	*A*_*r*_	*H*_*o*_	*H*_*e*_	*F*_*is*_	*N*_*e*_	*N*_*e*_ 95% CIs
Ash Creek^a^	P1	53	1.67	0.19	0.20	0.04	26	20–35
Byrds Creek	B1	44	1.57	0.15	0.19	0.17	31	24–40
Fancy Creek	P4	42	1.75	0.21	0.21	0.02	65	39–148
Gault Hollow	P5	32	1.74	0.18	0.21	0.11	114	41– ∞
Harker Creek	O1	47	1.73	0.17	0.20	0.14	128	67–639
Horse Creek	P2	48	1.73	0.18	0.21	0.11	48	37–65
Lawrence Creek^b^	MR1	50	1.74	0.19	0.22	0.13	535	231–∞
Lowery Creek^a^	SG1	50	1.73	0.20	0.21	0.04	79	53–139
Marshall Creek	P3	43	1.79	0.21	0.22	0.03	54	38–87
Melancthon Creek^a^	P6	50	1.64	0.19	0.19	0.00	45	31–70
South Fork Hay River^a^	SF1	50	1.75	0.19	0.20	0.06	294	145–5556
Upper Pine Unnamed Tributary	P7	46	1.68	0.16	0.20	0.17	20*	15–29*
West Branch Mill Creek^a^	MC1	39	1.74	0.20	0.20	0.04	89	56–185
St. Croix Falls Hatchery strain	SCF	49	1.74	0.19	0.21	0.09	137	82–338

* Effective population size estimate is likely biased low due to recent admixture and subsequent elevated linkage between loci.

^a^ This population has or is currently being used as a locally derived broodsource.

^b^ This population has been used as a wild source for translocations.

### 2.3 Library Preparation

Libraries were prepared following the BestRAD protocol (Ali et al. 2016). Total genomic DNA was extracted using the Promega Wizard Genomic DNA purification kit (Promega Corporation, Madison, Wisconsin). DNA concentrations were estimated using Quant‐iT dsDNA PicoGreen assays (ThermoFisher Scientific, Waltham, Massachusetts) and a BioTek Synergy HTX Plate Reader (Agilent Technologies, Santa Clara, California). Approximately 100 ng of each sample was digested in 2 μL reactions with restriction enzyme *SbfI* (New England Biolabs, Ipswich, Massachusetts). Barcodes were then ligated to digested samples and sheared to fragment lengths of 300‐500 bp using 12–14 30‐s cycles of a Q500 sonicator (Qsonica, Newtown, Connecticut). These fragments were subsequently bound to Dynabeads M‐280 Streptavidin magnetic beads (ThermoFisher Scientific, Waltham, Massachusetts) and washed to remove non‐target sequences. We then size‐selected these fragments using AMPure XP beads (Beckman Coulter, Brea, California) targeting an insert size of 250 bp and enriched our libraries following the NEBNext Ultra DNA Library Prep Kit for Illumina (New England Biolabs, Ipswich, Massachusetts) using plate‐specific i7 barcodes and 12 cycles of PCR. Enriched and size‐selected libraries were visually confirmed on a 2% agarose E‐Gel (ThermoFisher Scientific, Waltham, Massachusetts) before a final AMPure XP bead purification. Final libraries were quantified using Quant‐iT dsDNA PicoGreen assays and pooled to equimolar concentrations prior to sequencing.

### 2.4 Sequencing, Genotyping, and Filtering

Sequencing was conducted on an Illumina Novaseq 6000 using 1.4 lanes of 2 × 150bp S4 chemistry (Novogene, Sacramento, California). Raw reads were processed using stacks v2.3 (Catchen et al. 2011, 2013). Briefly, sequences were demultiplexed by i5 and i7 barcodes, filtered for *SbfI* restriction sites, and trimmed to lengths of 140 bp (i.e., ‐e SbfI ‐c ‐q ‐r ‐t 140 ‐‐filter_illumina ‐‐bestrad). Individual‐specific reads passing these filters were aligned with ustacks and parameters previously shown to avoid under‐ and over‐merging loci from salmonid RAD datasets (i.e., ‐‐disable‐gapped ‐m 3 ‐M 5 ‐H ‐‐max_locus_stacks 4 ‐‐model_type bounded ‐‐bound_high 0.05; Ackiss, Larson, and Stott 2020). A catalog of loci was then built using all resultant stacks and the cstacks module (‐n 3 ‐p 6 ‐‐disable_gapped). Individual‐specific stacks were then matched against the locus catalog using the sstacks module (‐‐disable_gapped). Paired end reads were then imported and all data were transposed by locus using tsv2bam. Paired end contigs were assembled and SNPs were called with the gstacks module. Resultant individual genotypes were exported in variant call format for loci genotyped in at least 30% of individuals using the populations module (−*r* 0.3). We further used vcftools v0.1.16 to filter this dataset by sequentially removing loci with minor allele counts less than three, loci that were not successfully genotyped in ≥ 70% of individuals in each population, and individuals that were not genotyped at ≥ 50% of loci (‐‐mac 3, ‐‐exclude‐positions, and ‐‐remove, respectively; Danecek et al. 2011). Paralogous loci, resultant from a recent whole genome duplication event in salmonids, were identified and removed using HDPlot with absolute read deviations > 8 and observed heterozygosity > 0.5 (McKinney et al. 2017). Finally, as some of our downstream analyses assume independence of loci, we further filtered this dataset by retaining only one SNP per contig and chose to retain the SNP with the highest minor allele frequency in these instances.

### 2.5 Genetic Diversity

Allelic richness (*A*_*r*_), observed heterozygosity (*H*_*o*_), expected heterozygosity (*H*_*e*_), and inbreeding coefficients (*F*_*IS*_) were calculated for each sampling location, which we hereafter consider as populations, using the R package “diveRsity” v1.9.90 (Keenan et al. 2013). Effective population sizes (*N*_*e*_) and associated 95% jackknifed confidence intervals were estimated using the single‐sample linkage‐disequilibrium‐based method in NeEstimator v2.1 and a minimum allele threshold of 0.02 (Waples and Do 2008, 2010; Do et al. 2014; Jones, Ovenden, and Wang 2016). However, because this method assumes independence of loci, we aligned the contigs from a previously established brook trout linkage map (Hale et al. 2017) to our contigs to determine chromosomal locations and subsequently exclude comparisons between loci located on the same linkage group. Logarithmic relationships between genetic diversity metrics and effective population sizes were evaluated with modified linear regressions in base R (R Core Team 2022).

### 2.6 Population Structure, Gene Flow, and Hatchery Introgression

Population structure was assessed through a combination of pairwise *F*_*ST*_ estimates, neighbor‐joining trees, principal component analysis (PCA), and isolation‐by‐distance (IBD) analysis. Pairwise *F*_*ST*_ estimates were calculated using the “diveRsity” v1.9.90 package, neighbor‐joining trees were constructed using Nei's genetic distance and 1000 bootstrap replicates in PopTree2 and visualized using the “ggtree” v3.4.1 package (Nei, Tajima, and Tateno 1983; Felsenstein 1985; Takezaki, Nei, and Tamura 2010; Yu et al. 2017), and PCA was conducted using the “adegenet” v2.1.7 package (Jombart 2008; Jombart and Ahmed 2011). Isolation‐by‐distance was assessed by regressing and permutating the linearized *F*_*ST*_ matrix against a corresponding waterway distance matrix calculated using the QNEAT3 v1.0.3 plugin implemented in QGIS v3.10.4, base R functions, and the “ade4” v1.7–19 package (Chessel, Dufour, and Thioulouse 2004; QGIS Development Team 2022). Note that we excluded Lawrence Creek from IBD analysis as this population is located in the Great Lakes watershed and would therefore need to migrate thousands of kilometers to interact with the Mississippian populations in our dataset. Gene flow and hatchery introgression were assessed using Bayesian clustering analysis implemented in ADMIXTURE v2.3 (Alexander, Novembre, and Lange 2009) with numbers of clusters (i.e., ancestral populations; *K*) values ranging from 1 to 20 and fivefold cross validation.

### 2.7 Drift Simulations

Preliminary investigations of population structure, gene flow, and hatchery introgression indicated that our sampled populations were surprisingly divergent given their hydrological proximity, patterns of divergence did not follow watershed boundaries, natural gene flow was minimal, and wild populations did not show clear signs of introgression with the St. Croix Falls Hatchery strain. Accordingly, we surmised that our sampled populations might not be at drift‐migration equilibrium, and that observed levels of divergence may be due to rapid genetic drift from a recent common source.

Simulations are a useful method to assess the effects of genetic drift on ancestry estimation as they allow for control and flexibility of demographic and evolutionary parameters. To this aim, we utilized our empirical effective population size point estimates to conduct drift‐based simulations to examine our power to reassign individuals back to a common source over varying evolutionary timescales. All simulations were conducted using EASYPOP v2.01 (Balloux 2001) and consisted of 13 diploid populations analogous to our sampled wild populations and the St. Croix Falls hatchery strain. Population sizes were parameterized with empirical effective population size point estimates for each population. Note that we excluded an analog (simulated population) corresponding to the unnamed tributary to the Pine River from these simulations as the presence of hatchery introgression (discussed below) upwardly biases linkage disequilibrium and therefore we would not be able to accurately parameterize the effective size of this population. All populations started with the same allele frequencies (i.e., *H*_*o*_ = 0.5) for 500 independent and neutral biallelic loci that did not undergo mutation. Similarly, as our objective was to investigate the effect of drift on divergence and ancestry estimation under modern conditions of little population connectivity, we did not allow for gene flow between populations. We allowed populations to diverge for 0, 5, 10, 20, 30, 40, and 50 generations; conducted 100 replicates for each time step; and exported genepop‐formatted files for each simulation. Assuming an average generation time of 2 years, these simulations would encompass 100 years of divergence (Letcher et al. 2007; Wood, Welsh, and Todd Petty 2018). Note that while simulation parameters and analyses hereafter utilize parameterizations relating to the St. Croix Falls hatchery strain for the purposes of evaluating the accuracy of estimating historical hatchery introgression, these results would also be analogous to estimating historical contributions from any source (e.g., wild remnants) with comparable effective population sizes.

Simulation results were analyzed similarly to empirical data to determine the effect of drift on ancestry inference. First, we selected a random subset of 25 individuals from each population and converted these data to browser extensible data (.bed) format using the “radiator” v1.2.2 package (Gosselin 2020), PGDSpider v2.1.1.5 (Lischer and Excoffier 2012), and plink v1.90 (Purcell et al. 2007). Each subset replicate was then analyzed with ADMIXTURE v2.3 testing *K* values from 1 to 20 with fivefold cross validation. All runs were aligned and visualized with the package “pophelper” v2.3.1 (Francis 2017). To generalize the effect of drift on the divergence of populations and the accuracy of hatchery introgression, we first averaged Q‐scores (assignment probabilities) within populations and across replicates for *K* = 13 models. We then constructed nested beta regressions to determine the relationship between effective population sizes and divergence timelines (i.e., number of generations) to assignment probabilities corresponding to the eventually distinct cluster associated with each wild population. Likewise, we determined the relationship between effective population size and divergence timelines to assignment probabilities associated with the simulated hatchery strain. In this sense, we seek to model the amount of time required for populations of varying size to appear as distinct and the increasing dissimilarity to the simulated hatchery strain over time, respectively. The former scenario would represent a situation where two populations derived from a common source would be misattributed as two distinct, and potentially remnant, populations while the latter scenario would represent increasing underestimation of hatchery introgression (or shared ancestry from another common source) over time.

## 3 Results

### 3.1 Sequencing, Genotyping, and Filtering

We obtained a total of 5,132,574,726 reads corresponding to our 672 barcoded samples. The number of retained reads per individual ranged from 11,740 to 40,863,005 with an average and median of 7,576,710 and 6,369,840 reads, respectively. These reads were used to identify 38,399 SNPs after filtering out loci that were not genotyped in at least 70% of individuals in all populations. We further screened these loci for paralogs and removed 2167 loci with evidence of duplication. The remaining 36,216 singletons were filtered to retain only one SNP per contig which yielded a final set of 21,541 SNPs. We also removed 29 individuals (4.3%) that were not genotyped at > 50% of loci. The remaining 643 individuals were successfully genotyped at a mean and median of 93.0% and 97.1% of loci, respectively (range = 49.9%–99.2%).

### 3.2 Genetic Diversity

Wild population estimates of allelic richness, observed heterozygosity, and expected heterozygosity ranged from 1.569–1.786 (mean = 1.710), 0.154–0.214 (mean = 0.187), and 0.187–0.221 (mean = 0.205), respectively (Table 1). The St. Croix Falls strain exhibited similar levels of diversity with estimates of allelic richness, observed heterozygosity, and expected heterozygosity of 1.736, 0.187, and 0.209, respectively (Table 1). Linkage disequilibrium estimates of contemporary effective population size were highly variable and ranged from 20 to 535 (mean = 118) for wild populations, while the St. Croix Falls hatchery strain was estimated to be 137 individuals (Table 1). Inbreeding coefficient estimates ranged from 0.004–0.168 (mean = 0.081) for wild populations and was 0.093 for the St. Croix Falls strain (Table 1). No significant correlations (*p* > 0.05) were observed between metrics of genetic diversity and effective population size point estimates (Figure S1).

### 3.3 Population Structure

**FIGURE 2:**
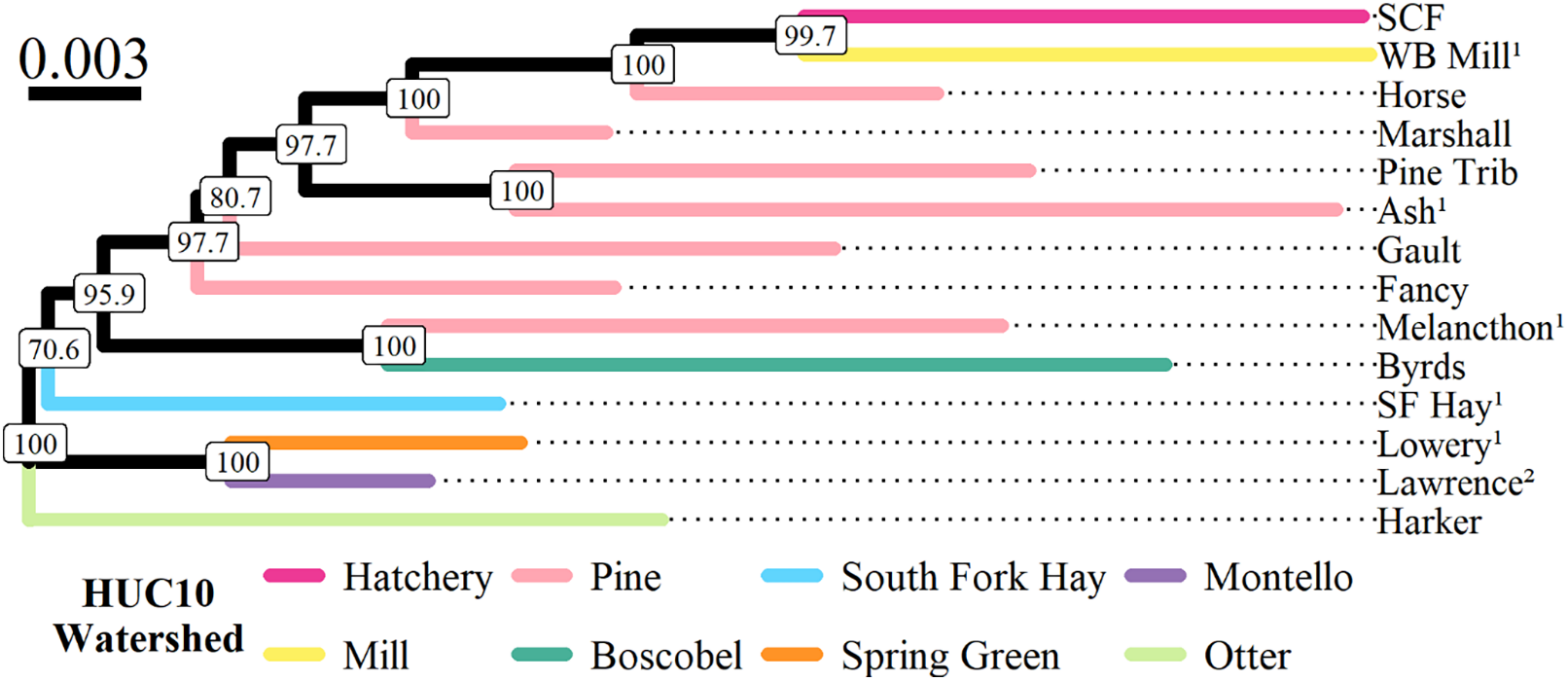
Neighbor‐joining tree of Nei's genetic distance (*D*_*A*_) for 13 wild brook trout populations from Wisconsin and the St. Croix Falls hatchery strain. Branches are color‐coded by HUC10 drainage and follow the same scheme as the geographical maps in Figure 1. Node labels represent bootstrap values from 1000 replicates. Superscripts denote populations used as wild broodsources (^1^) or translocation sources (^2^).

**FIGURE 3:**
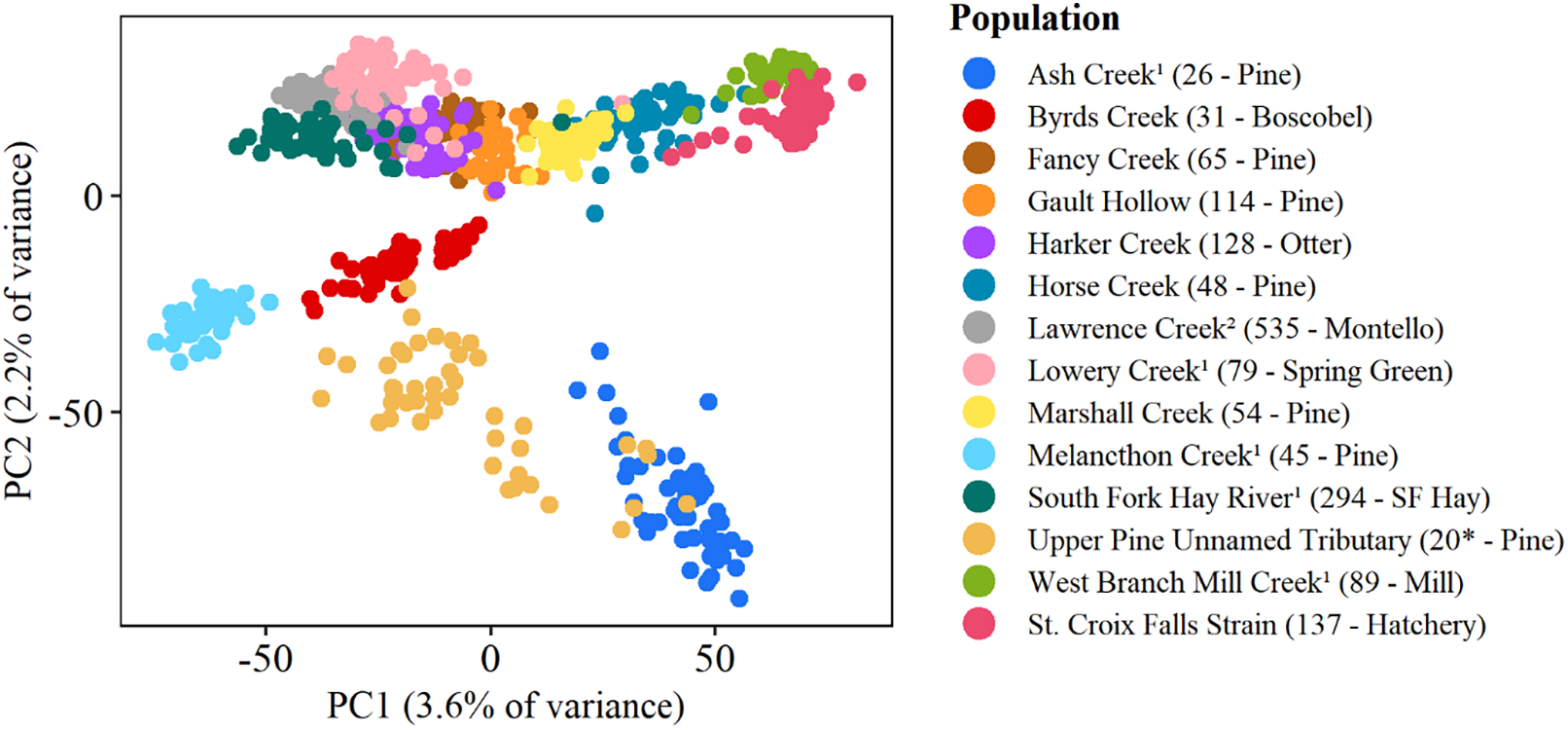
Principal components analysis of 13 wild brook trout populations from Wisconsin and the St. Croix Falls hatchery strain. Linkage disequilibrium‐based effective population size point estimates are provided in parentheses and asterisks denote estimates that are likely biased low due to recent introgression. HUC10 watersheds are also indicated in parentheses and superscripts denote populations used as wild broodsources (^1^) or translocation sources (^2^).

**FIGURE 4:**
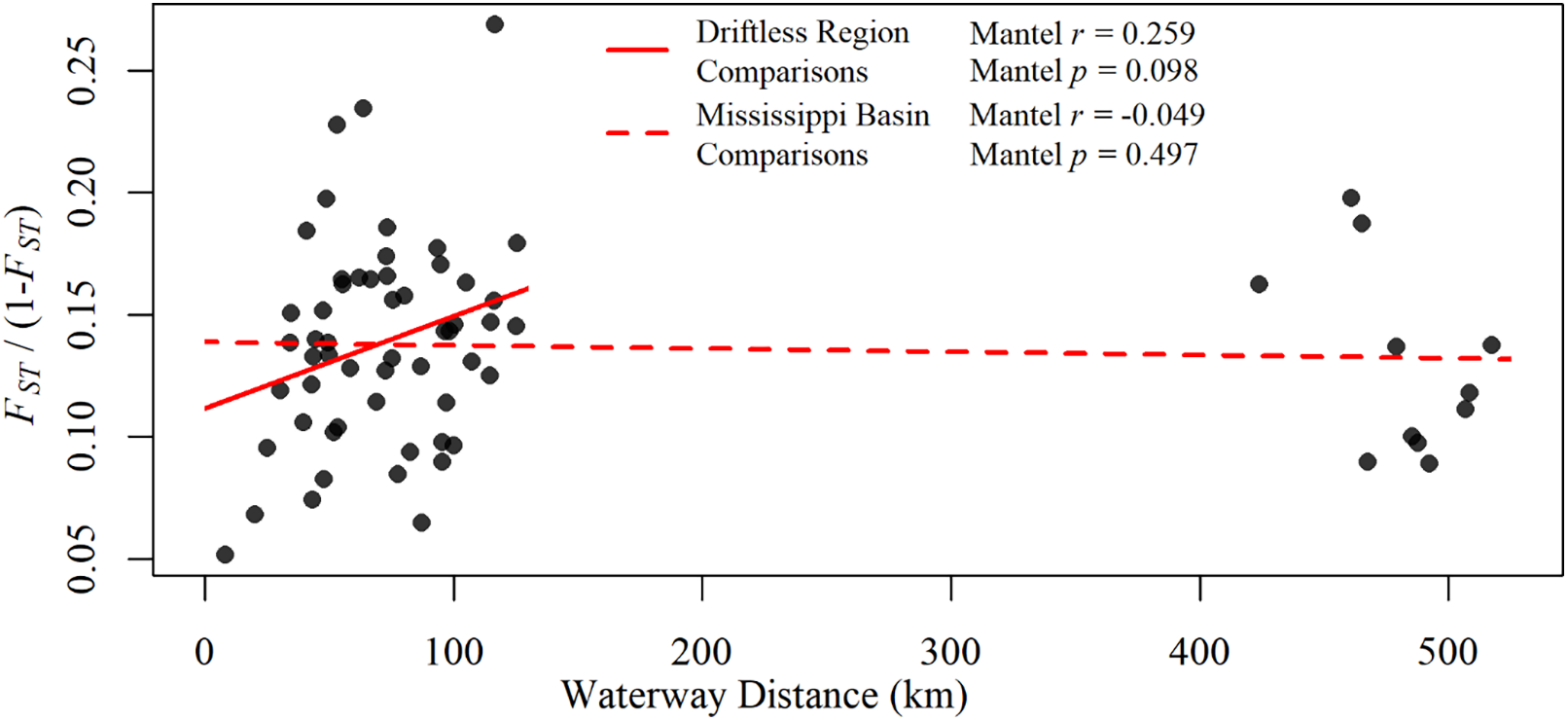
Brook trout populations analyzed by this study do not exhibit significant isolation‐by‐distance. This is evident when observing only populations in the Driftless Area (solid line) or the greater Mississippi River basin (dashed line).

Most populations showed moderately high levels of divergence (overall *F*_*ST*_ = 0.118, range = 0.034–0.212). Comparisons of wild populations to the St. Croix Falls strain showed similar levels of divergence as the full dataset (average *F*_*ST*_ = 0.134, range = 0.073–0.205). These results are congruent with the topology of our neighbor‐joining tree as evidenced by long branch lengths and relatively short distances between nodes (Figure 2). Hydrological population structure was largely absent in our neighbor‐joining tree topology and PCA clustering patterns (Figures 2 and 3, respectively), and IBD analyses corroborated these findings with no significant relationship (*p* > 0.05) between hydrological proximity and genetic differentiation (linearized *F*_*ST*_) of populations in the Driftless Area and greater Mississippi Basin (i.e., including the South Fork of the Hay River; Figure 4). Collectively, these findings suggest that brook trout populations in the Driftless Area are substantially divergent from one another but do not exhibit strong hydrological population structure as found with native populations elsewhere in their range.

**FIGURE 5:**
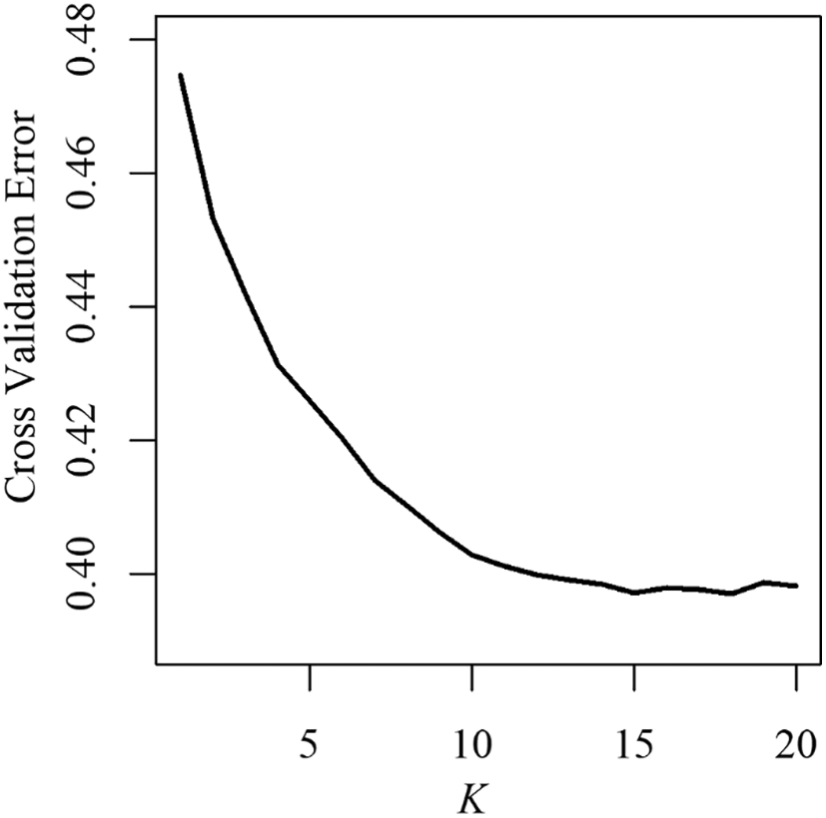
Cross validation error over increasing numbers of ancestral clusters (*K*) for 13 wild brook trout populations and the St. Croix Falls Hatchery strain. These errors are minimized at *K* = 18. However, these results do not show any discernable substructuring in our sampled populations and we accordingly show results of *K* = 14 in Figure 6 as this number corresponds to the number of sampled populations and results in distinct clusters for each population.

**FIGURE 6:**
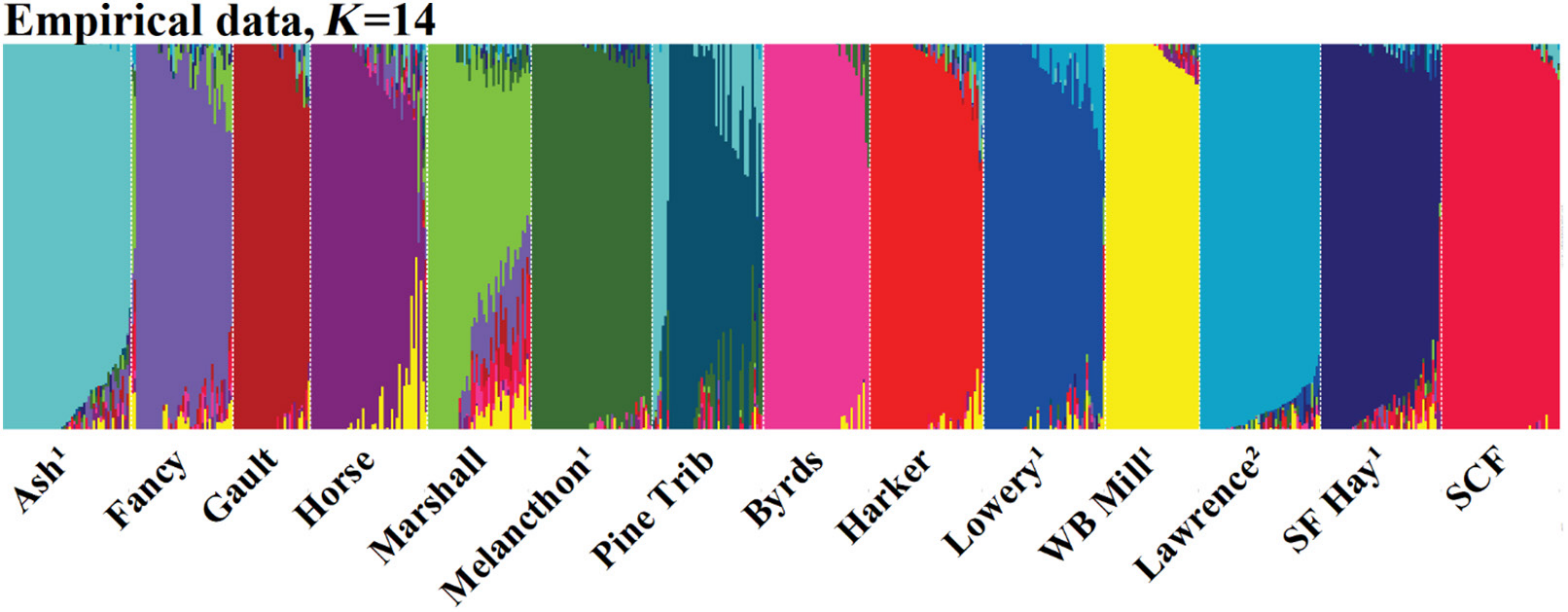
Results of empirical ADMIXTURE results using 14 ancestral clusters (*K*). Superscripts denote populations used as wild broodsources (^1^) or translocation sources (^2^). Populations predominantly assign to distinct clusters and admixture is largely absent, suggesting that streams harbor genetically unique populations. Introgression of the St. Croix Falls Hatchery strain (SCF) is minimal despite intensive stocking of this strain since 1972. However, there are some instances of introgression (i.e., Pine Trib, Lowery, Marshall) that may be explained by stocking of locally derived strains, wild translocations, hydrological proximity, and combinations thereof (see discussion for further expansion of these instances).

Cross‐validation of ADMIXTURE results suggest that the most supported number of ancestral clusters was higher than the number of sampled populations (*N* = 14; Figure 5). However, *K* values greater than 14 did not elucidate any discernible substructure within our sampled populations and we accordingly present results for *K* = 14 instead (Figure 6; see Figure S2 for graphical representation of all *K* values). The results generated using 14 ancestral clusters suggest that each stream harbors a genetically unique population, and that the St. Croix Falls strain is divergent from wild populations (Figure 6). Clustering analyses also suggested a lack of hatchery introgression in most populations as most wild individuals displayed very low assignment to known hatchery strains (Table S1; Figure 6). The one exception to this trend was the unnamed tributary to the Upper Pine River which displayed an unusually high introgression estimate of 21% attributed to a locally derived hatchery strain (Ash Creek; Table S1; Figure 6). The highest proportion of average assignment to the St. Croix Falls strain was 5% (Table S1). Assignment to locally derived hatchery strains (except for the unnamed tributary described above) and wild translocation sources was similarly low (Table S1; Figure 6).

### 3.4 Drift Simulations

**FIGURE 7:**
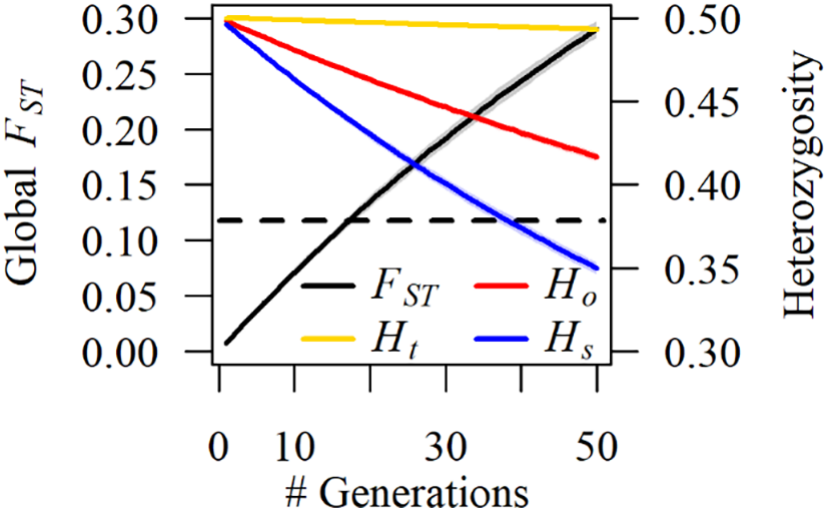
Genetic differentiation (global *F*_*ST*_), expected global heterozygosity (*H*_*t*_), observed global heterozygosity (*H*_*o*_), and observed local heterozygosity (*H*_*s*_) ± two standard deviations for empirically informed simulations of genetic drift over time. The dashed line represents an empirical global *F*_*ST*_ estimate of wild populations. Note that these simulations only model drift and do not include mutation or gene flow. Collectively, these results suggest increasing differentiation, sharply decreasing heterozygosity of local populations, and more stable global allele frequencies over time.

Drift simulations showed expected patterns of increasing differentiation over time, with global *F*_*ST*_ approaching our empirical estimate of *F*_*ST*_ in approximately 17 generations (Figure 7). Observed heterozygosity declined rapidly in these simulations and was more pronounced at the population‐level (Figure 7). In contrast, expected global heterozygosity was relatively stable, consistent with expectations of drift randomly affecting loci at the population‐level (Figure 7).

**FIGURE 8:**
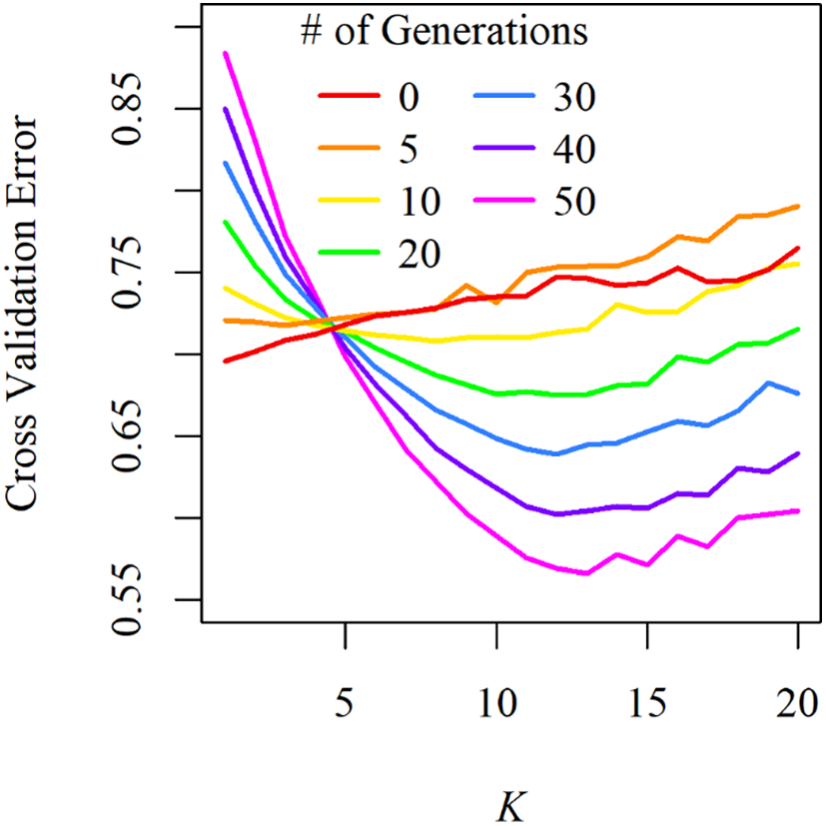
Cross validation error of increasing numbers of ancestral clusters (*K*) for empirically informed simulations of genetic drift over time. Each colored curve represents the average from 100 simulation replicates and standard errors have been omitted as they are functionally zero (SD < 0.0000). Best supported numbers of ancestral clusters, as evidenced by minimized errors of the curves, range from *K* = 1 for zero generations of drift (due to approximately equal allele frequencies among all populations) to *K* = 13 (i.e., the number of simulated populations) after 50 generations of drift. These values increase over time (up to *K* = 13) as variability in effective population sizes results in variable rates of divergence that necessitate more time for detectable differences to accrue in larger populations (see Figures 9 and S3 for demonstration of this phenomenon).

**FIGURE 9:**
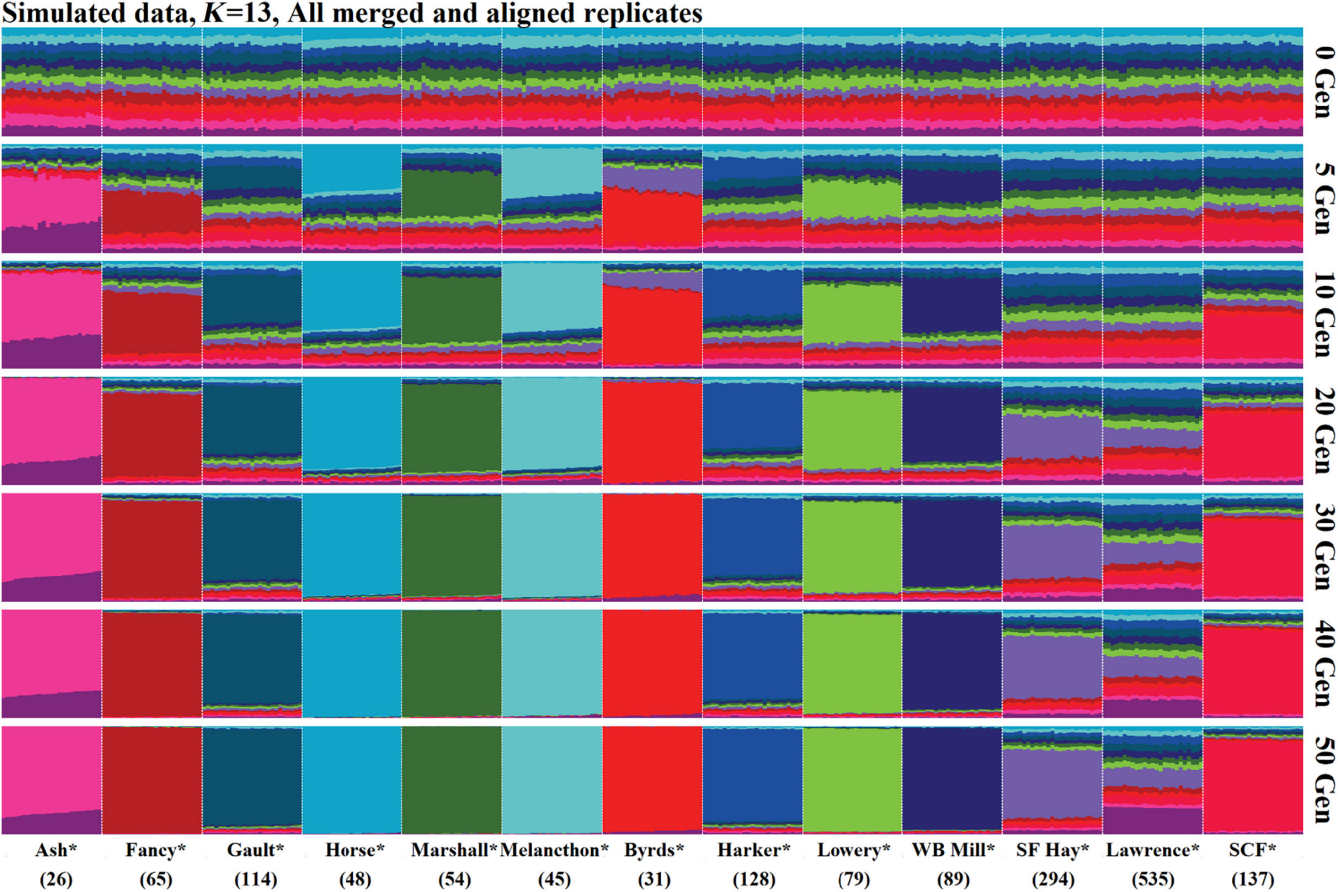
ADMIXTURE results using 13 ancestral clusters (*K*) for empirically informed simulations of genetic drift over time (i.e., generations; denoted to the right of each plot). Simulated populations are labeled by their empirically informed analogs and their effective population sizes are denoted in parentheses. Note that population structure is initially absent at zero generations as populations start with approximately equal allele frequencies (plus or minus sampling error) and populations with lower effective population sizes diverge more quickly. Most populations assign to distinct clusters after 20–30 generations of drift.

ADMIXTURE analysis of these simulations also clearly demonstrated the increasing differentiation of populations over time. This trend can be easily visualized by examining the cross‐validation error curves of increasing divergence timelines (Figure 8). For example, the best‐supported number of ancestral clusters is one after zero generations of drift (i.e., when populations start with approximately equal allele frequencies) and increases to 13 (the number of simulated populations) after 50 generations of drift (Figure 8). Aligning *K* = 13 plots for each divergence timeline makes this pattern readily apparent as variable effective population sizes result in rapid differentiation of small populations and longer timescales required for distinguishable differences to accrue in larger populations (Figure 9). While examining these patterns is most intuitive using *K* values corresponding to our number of simulated populations, we acknowledge that forcing populations into a less supported number of clusters can be inadvisable for empirical data, and we accordingly provide results using the best supported number of clusters for each divergence timeline (Figure S3).

**FIGURE 10:**
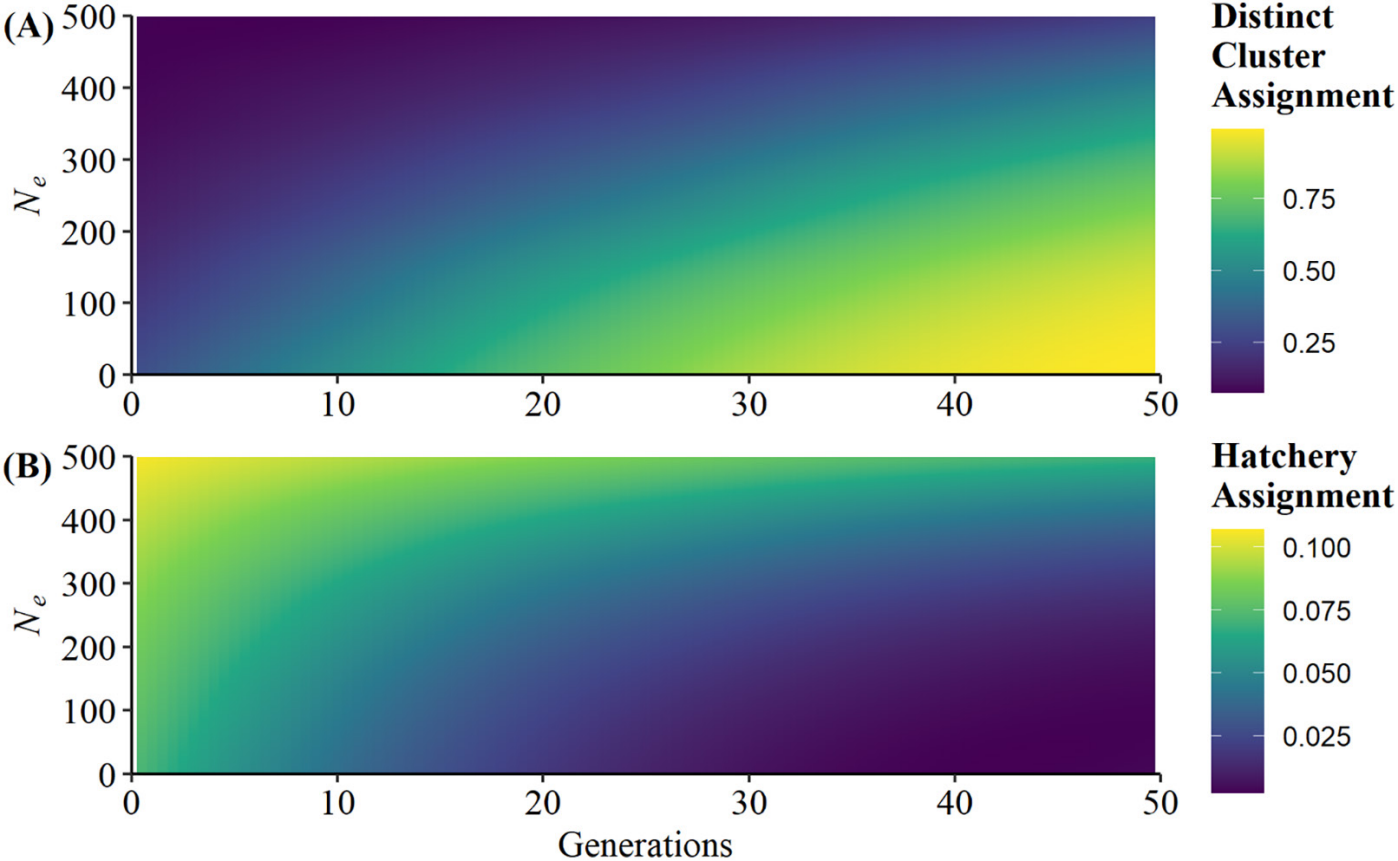
(A) Predictions of average assignment of wild population analogs to their eventual distinct clusters (e.g., Ash and Fancy to pink and maroon clusters, respectively, in Figure 9) in empirically informed genetic drift simulations. Predictions were made using the top performing model from Table 2. (B) Predictions of average assignment of wild population analogs to the predominant cluster associated with a hatchery strain analog in drift simulations. Predictions were made using the top performing model from Table 3. Note that the scales on panels (A) and (B) differ by an order of magnitude.

Beta regressions of ADMIXTURE results also supported the rapid differentiation of populations and reassignment back to hatchery strains decreasing over time (Tables 2 and 3). Effective population size, number of generations of drift, and an interaction of these variables were included in best‐supported models for each scenario (Tables 2 and 3). This follows the expectation that divergence is greater in small populations and over long timescales, and that this divergence leads to assignment of distinct clusters rather than back to a common source. These inferences are consistent with Figures 9 and S3 where each population initially exhibits approximately equal assignment to all ancestral clusters, populations with *N*_*e*_ < 100 start to assign to a distinct dominant cluster by as early as 5 generations, and populations with *N*_*e*_ < 300 largely assign to their own distinct cluster after 20–30 generations of divergence (Figure 10A). Consequently, these models also suggest that ancestry may be obscured in wild populations experiencing rapid drift, and that the underestimation of these historical contributions (either from hatchery sources or remnant wild populations) is likely to be exacerbated with increasing divergence timelines and lower effective population sizes (Figure 10B). For example, assignment of wild populations to the cluster eventually associated with the hatchery strain analog is approximately equal to the reciprocal of the number of populations (i.e., 1/13 = 0.077) after zero generations of drift and decreases over time, with more pronounced declines in small populations (Figure 10B).

**TABLE 2:** Relative likelihoods and parameter coefficient estimates (*β*) for beta regression models predicting the average assignment of wild population analogs to their eventual distinct clusters (e.g., Ash and Fancy to pink and maroon clusters, respectively, in Figure 9) in empirically informed genetic drift simulations. Degrees of freedom (df), log‐likelihoods (LL), second‐order Akaike information criterion (AIC_*c*_), differences in AIC_*c*_ scores between candidate models and the best performing model (∆AIC_*c*_), Akaike weights (*w*), and measures of fit relative to the null model (Psuedo‐*R* ^2^) are provided. Predictions from the best performing model (top row) are visualized in Figure 10A.

Intercept (*β*_0_)	# Gen (*β*_1_)	*N*_*e*_ (*β*_2_)	# Gen:*N*_*e*_ (*β*_3_)	df	LL	AIC_*c*_	∆AIC_*c*_	*w*	Psuedo‐*R* ^2^
−0.8904	0.1004	−0.0039	−0.0001	5	101.5	−192.3	0.0	1.00	0.863
−0.5227	0.0841	−0.0077	—	4	94.2	−179.9	12.4	0.00	0.829
−1.0894	0.0649	—	—	3	43.9	−81.6	110.7	0.00	0.597
0.8200	—	−0.0042	—	3	15.1	−23.9	168.4	0.00	0.235
0.2573	—	—	—	2	4.0	−3.8	188.5	0.00	—

**TABLE 3:** Relative likelihoods and parameter coefficient estimates (*β*) for beta regression models predicting the average assignment of wild population analogs to the predominant cluster associated with a hatchery strain analog in empirically informed genetic drift simulations. Degrees of freedom (df), log‐likelihoods (LL), second‐order Akaike information criterion (AIC_*c*_), differences in AIC_*c*_ scores between candidate models and the best performing model (∆AIC_*c*_), Akaike weights (*w*), and measures of fit relative to the null model (Psuedo‐*R* ^2^) are provided. Predictions from the best performing model (top row) are visualized in Figure 10B.

Intercept (*β*_0_)	# Gen (*β*_1_)	*N*_*e*_ (*β*_2_)	# Gen:*N*_*e*_ (*β*_3_)	df	LL	AIC_*c*_	∆AIC_*c*_	*w*	Psuedo‐*R* ^2^
−2.4686	−0.0823	0.0006	0.0002	5	286.7	−562.7	0.0	1.00	0.702
−2.7596	−0.0515	0.0024	—	4	257.4	−506.3	56.4	0.00	0.593
−2.3539	−0.0561	—	—	3	242.7	−479.1	83.7	0.00	0.448
−3.8078	—	0.0035	—	3	214.6	−422.9	139.8	0.00	0.164
−3.2348	—	—	—	2	199.9	−395.7	167.0	0.00	—

## 4 Discussion

Brook trout populations in the Driftless Area of Wisconsin have undergone a complex history of habitat degradation and hatchery supplementation that has resulted in uncertainty surrounding whether contemporary populations possess remnant native or hatchery ancestry. Genomic data suggest that our wild study populations are substantially divergent even within relatively small watersheds, likely due to small effective population sizes and limited gene flow among populations separated by as little as a few kilometers. Notably, these populations do not show hydrological population structure or isolation‐by‐distance patterns characteristic of native populations elsewhere in their range (Lehnert et al. 2020; Morgan et al. 2021; Erdman, Gallagher, et al. 2022; Kazyak et al. 2022). However, they also are substantially divergent from known hatchery sources. At face value, these results would often be interpreted as introgression or founding events from uncharacterized sources, but we show through empirically informed simulations that observed levels of divergence can be produced by genetic drift over timescales comparable to stocking histories. Collectively, these results suggest that it is difficult to rule out that many populations in the Driftless Area may have been historically extirpated and refounded, and that commonly used methods to estimate hatchery introgression may be confounded by rapid genetic drift.

### 4.1 Genetic Diversity

Populations analyzed in this study showed relatively little variability in allelic richness and heterozygosity estimates despite considerable variability of effective population size point estimates (Table 1). Furthermore, the St. Croix Falls strain exhibited observed and expected heterozygosity estimates consistent with the mean of wild populations and slightly higher allelic richness and effective population size estimates (Table 1). Effective population size estimates were also substantially lower for our study populations proximate to the Pine River Watershed (mean = 64, range = 26–128) than the two other populations located in other regions of Wisconsin (mean = 415, range = 294–535). No significant relationships between genetic diversity metrics and effective population size were observed, suggesting that gene flow (either natural or artificial) may be obscuring these relationships or that populations might not yet be at drift‐migration equilibrium (Figure S1).

### 4.2 Population Structure, Gene Flow, and Hatchery Introgression

Our findings support previous research suggesting high divergence and a lack of strong hydrological population structure for wild brook trout populations in Wisconsin (Hughes 2008; Erdman, Mitro, et al. 2022). Such high levels of divergence between hydrologically proximate populations in this study were unexpected, as we initially surmised that these populations would be most likely to exchange gene flow. In fact, we designed this study to identify the scale of gene flow in Wisconsin brook trout and hypothesized that populations separated by a few kilometers were extremely likely to exchange at least some migrants. Yet, we observed very few migrants or F_1_ hybrids between populations, suggesting that natural gene flow is minimal even within relatively small stream networks in this region (Figure 6). The reasons behind this lack of gene flow are not well understood but may be partially explained by the confinement of populations to spring‐fed headwater reaches in the Driftless Area that is reinforced by the downstream presence of competing species (i.e., brown trout, *Salmo trutta*), seasonably unsuitable habitat conditions in higher order streams, physical barriers, natal homing, or local adaptations (Hoxmeier and Dieterman 2013; Olson, Mauel, and Pechacek 2021; Olson 2023). Regardless of the mechanism, it is clear that populations in our study region are largely genetically isolated, and that many populations may not yet be at drift‐migration equilibrium, if they exchange migrants at all.

The results of ADMIXTURE analysis suggest a substantial lack of hatchery introgression in most populations. This was rather surprising considering previous reports of widespread extirpations in the region and the subsequent rebounding of populations following intensive habitat remediation and stocking (Greene 1935; Becker 1966, 1983; Trimble and Lund 1982; Fago 1992; Thorn et al. 1997; Vetrano 2017). Indeed, decades of stream surveys suggest that brook trout were almost entirely extirpated from the Pine River watershed before the establishment of the St. Croix Falls strain in 1972, and yet the highest average assignment probability to this strain was only 5.4% (Table S1; Figure 6; Greene 1935; Becker 1983; Fago 1992; but see Becker 1966 for reports of three small populations). Contributions from locally derived hatchery strains and translocation sources were similarly low except for one high estimate of 20.7% attributed to Ash Creek in an unnamed tributary to the Upper Pine River (Table S1; Figure 6). Interestingly, this unnamed tributary has no documented stocking history and is located 72.6 km from Ash Creek, but 9229 Ash Creek adults were stocked in two nearby streams from 2013 to 2017. The presence of pure Ash Creek individuals, F_1_ hybrids, and wild backcrosses in the unnamed tributary suggests that stocked trout can, in some cases, emigrate from their recipient streams and successfully reproduce in adjoining waterbodies (Figure 6). This is somewhat paradoxical considering the lack of natural gene flow observed in our data but may be explained by closer proximity and the distal position of these populations relative to the confluence of the river mainstem, increased straying rates of hatchery progeny, or competition from pre‐existing brook trout populations (Figure 1; Quinn 1993; Keefer and Caudill 2014; Olson, Mauel, and Pechacek 2021). The former hypothesis is further supported by additional Melancthon Creek backcrosses in the unnamed tributary to the Upper Pine River and higher prevalence of hybrid backcrosses between the most hydrologically proximate stream pair in our dataset (Fancy and Marshall creeks; Figures 1 and 6). However, it appears that this pattern is affected by additional factors other than hydrological distance as isolation‐by‐distance relationships were insignificant, both in the Driftless Area and when including samples from the greater Mississippi River basin (Figure 4). Future research should include ecological and environmental variables (e.g., water temperature, pre‐existing fish assemblages, etc.) into spatial analyses of genetic differentiation such that they represent isolation‐by‐resistance, rather than the more naïve isolation‐by‐distance, to elucidate the factors modulating isolation within stream networks.

### 4.3 Drift Simulations

Empirically informed simulations of genetic drift suggest that discernable differences between populations may accrue within a few decades. Indeed, it took only 17 generations for simulation global *F*_*ST*_ estimates to reach levels consistent with our empirically derived estimate, and by this time most populations with effective sizes less than 300 individuals showed predominantly distinct profiles (Figures 7, 9, and S3). Such short timescales of divergence from a common wild or domestic source may be particularly problematic for accurately inferring ancestry as stocking histories and habitat remediation often span long time periods (e.g., several decades or even centuries; Wisconsin Fish Commission 1877). Accordingly, rapid drift‐based divergence may result in misattributing hatchery‐founded or recently recolonized populations as potentially distinct native remnants (Figure 10B). It is also important to note that this drift process is not restricted to the divergence of wild populations but simultaneously acts in hatchery strains, which again often have modest effective population sizes (e.g., Kazyak et al. 2022). Drift of hatchery strains may be especially troublesome if historical hatchery samples are not available as comparisons to modern samples may result in chronic underestimation of historical introgression that is also exacerbated with time since stocking. Indeed, this phenomenon may not be restricted to nominal hatchery strains, but even different hatcheries where a common “strain” is maintained without gene flow from other hatcheries.

It is not yet clear how to best disentangle recent, rapid genetic drift from slow genetic drift that has accumulated centuries or millennia. However, we suggest that haplotype information may help address these challenges through comparisons of linkage between syntenic alleles at varying distances. Under such a scenario, it would be expected that there would be stronger linkage between distant syntenic alleles in a population undergoing recent genetic drift compared to slow genetic drift, due to a lower number of recombination events. Likewise, understanding rates of linkage in relation to chromosomal locations would allow for the reconstruction of historical effective population sizes, temporal rates of drift, and attempts to resolve demographic and evolutionary scenarios through more advanced simulation‐based approaches (e.g., approximate Bayesian computation; Corbin et al. 2012; Nadachowska‐Brzyska, Konczal, and Babik 2022).

The goal of our simulations was not to provide a comprehensive overview of the demographic processes affecting the accuracy of hatchery introgression estimates. Rather, it was to provide a demonstration of how challenging it can be to exclude contemporary genetic drift as a major contributor to apparent population divergence among both hatchery and wild stocks. We hope these findings will serve as an impetus to investigate additional factors affecting the accuracy of hatchery introgression estimates. To this aim, we suggest several additional parameters that may affect these estimates. Additional factors expected to have the greatest effect on the accuracy of hatchery introgression estimates include gene flow, variable effective population sizes, and selection. Gene flow among wild populations will likely mitigate underestimation of introgression as divergence between populations will be reduced. In contrast, fluctuating effective population sizes will likely further obscure hatchery introgression as the magnitude of drift corresponds to the harmonic mean of effective population sizes that is more sensitive to bottlenecks than proliferation (Wright 1938; Kalinowski and Waples 2002). Likewise, selection is expected to contribute to the underestimation of introgression as selective sweeps reduce the frequency of maladaptive domesticated genotypes, effective population sizes are temporarily reduced, and environmental heterogeneity hastens divergence among populations. Mutation will also likely result in underestimation of introgression as divergence is increased, but this effect may be negligible over the relatively short timescales pertinent to stocking. Finally, increasing numbers of loci may also result in underestimation of introgression as the power to discriminate the emerging unique signatures of populations is increased.

### 4.4 Conservation Implications

A long and complex history of habitat degradation, remediation, and stocking has resulted in considerable uncertainty of the genetic origins of many Midwestern brook trout populations (Hughes 2008; Erdman, Mitro, et al. 2022). Previous research has shown a lack of broadscale hydrological population structure throughout the region coincident with high levels of differentiation between both wild populations and hatchery strains (Erdman, Mitro, et al. 2022). The results presented here corroborate these previous research efforts, extend them to extremely fine spatial scales, and provide further clarity of the demographic processes underpinning population structure in this region. Specifically, populations in the Driftless Area are often characterized by small effective population sizes, isolated to headwater reaches of watersheds, have low rates of natural gene flow, and are prone to rapid rates of genetic drift. Collectively, these results make it difficult to exclude the possibility that many populations in this region may have been historically extirpated and refounded by stocking events, translocations, or immigrants from remnant wild populations.

The historical extirpation of brook trout populations in the Driftless Area and subsequent reestablishment via stocking has been previously suggested based on the historical survey records and the absence of gill lice (*Salmincola* cf. *edwardsii*) in many contemporary populations (Fago 1992; Mitro and Griffin 2018). In short, this hypothesis suggests that gill lice, a regionally brook trout–specific parasite, would have been locally extirpated with their hosts following historical habitat degradation and contemporary lice‐free populations would therefore represent populations that were founded from lice‐free hatchery stocks and have remained isolated from other lice‐infected populations (Mitro and Griffin 2018). Indeed, many lice‐free populations are concentrated in the Driftless Area where extirpations were putatively widespread, stocking was intensive, and our genomic data suggests migration and gene flow between contemporary populations is minimal (Klingbiel 1975; Fago 1992; Mitro and Griffin 2018). Our empirically informed simulations of genetic drift further support this hypothesis as hatchery‐founded populations may approach our empirical observations of divergence in only 17 generations. Assuming the average brook trout generation length is approximately 2 years and considering the large increase in brook trout occupancy in our study region post‐1974, this timeline corresponds well to our sample collection dates (2015–2019), suggests many of our study population may be entirely hatchery‐founded, and the assumption of no gene flow in our simulations may be biologically accurate (Fago 1992; Letcher et al. 2007; Wood, Welsh, and Todd Petty 2018).

Unfortunately, the lack of any clear partitioning of allopatric lineages at broad spatial scales (i.e., Mississippian and Atlantic) prevented us from confidently delineating potentially remnant native populations from populations reestablished by stocking. To be clear, this does not mean that remnant adaptive genetic variation does not exist in the Driftless Area, just that recent population demography presents a significant challenge for discerning it with even large numbers of predominantly neutral markers. These difficulties do not necessarily preclude conservation of putative adaptive trait variation in brook trout from the Driftless Area or similar habitats (of which there are many for brook trout and other species). While it may prove difficult to clearly identify and preserve remnant native trout lineages in this region, the fact that many of these wild populations have persisted for decades in relative isolation raises the prospects that some may carry significant local adaptations to modern habitat conditions (Gordon et al. 2009). Such contemporary adaptations are difficult to ascertain outside of laborious experimentation (e.g., reciprocal translocation studies), but can have very large implications for population persistence and ecological function (e.g., Kinnison and Hairston 2007; Kinnison, Unwin, and Quinn 2008).

The results of our genetic drift simulations suggest that commonly used hatchery introgression estimation methods, based on clustering and assignment, may be prone to underestimate introgression in the face of rapid drift‐based divergence. These results are applicable not only to brook trout but any species that is characterized by low effective population sizes and limited gene flow. Indeed, these scenarios may be prevalent in the realm of hatchery introgression research as supplemental stocking is often used to increase abundance of depleted populations (Trushenski, Whelan, and Bowker 2018). In such instances, we urge others to take a more comprehensive approach that considers patterns of native population structure elsewhere in a species range, deviations from these patterns in observed data, and the scope of potential contemporary divergence corresponding to empirical estimates of effective population sizes and gene flow.

The potential of rapid divergence also raises questions about the mechanistic basis of recent claims that supplemented populations may be purging maladaptive hatchery alleles over relatively short timescales (Perrier et al. 2013; Harbicht, Wilson, and Fraser 2014; Valiquette et al. 2014; Létourneau et al. 2018; Erdman, Mitro, et al. 2022, Erdman, Gallagher, et al. 2022). These claims have largely been supported by evidence of increasing dissimilarity of contemporary wild populations and hatchery strains with increasing time since stocking (Valiquette et al. 2014; Létourneau et al. 2018; Erdman, Mitro, et al. 2022, Erdman, Gallagher, et al. 2022). However, in the absence of additional supporting information, such as from historical genetic samples, this pattern could also be produced by rapid genetic drift (with or without regional gene flow) and yet studies rarely rule out such a possibility. Resolving this question is of the utmost importance as the former suggests true recovery of native allele frequencies while the latter suggests the naturalization of a non‐indigenous lineage. Both scenarios could eventually result in the establishment of local adaptations, but the latter presents a potentially greater risk that deleterious hatchery alleles may persist longer in wild populations than implied by neutral divergence.

## 5 Conclusion

This study represents the first genomic investigation of brook trout population structure and hatchery introgression in the Driftless Area. These results suggest that populations in this region are often characterized by low effective population sizes, are confined to headwater reaches, and rarely exchange migrants. A lack of strong hydrological population structure and isolation‐by‐distance suggests that many populations may have been extirpated as a result of historical habitat degradation and subsequently refounded by stocking events or recent recolonization from remnant sources. Yet, wild populations remain substantially differentiated from each other and known hatchery strains, potentially as a result of rapid genetic drift. Empirically informed simulations of genetic drift suggest that these patterns could be generated over short timescales relevant to stocking histories. Such findings are particularly concerning as many investigations of hatchery introgression focus on species that exhibit low effective population sizes and may also be prone to rapid drift. We urge others to consider the effects of rapid drift on the accuracy of their ancestry estimates and to further explore additional factors (e.g., rates of gene flow, fluctuating effective population sizes, mutation) that may affect the accuracy of these estimates.

## Conflicts of Interest

The authors declare no conflicts of interest.

## Supporting information


Data S1.
**Table S1.** Summary of ADMIXTURE population‐specific average assignment probabilities to 14 ancestral clusters. Note that populations largely assign to their own distinct clusters. See Figure 6 for a graphical representation of the results used to calculate these averages.
**Figure S1.** Relationships of genetic diversity and inbreeding coefficients to effective population size for 12 wild brook trout populations and the St. Croix Falls Hatchery strain. Note that the unnamed tributary to the Upper Pine River was excluded from these analyses as its effective population size was unable to be accurately estimated due to recent introgression.
**Figure S2.** Results of empirical ADMIXTURE results using 1–20 ancestral clusters (*K*). Superscripts denote populations used as wild broodsources (^1^) or translocation sources (^2^).
**Figure S3.** ADMIXTURE results using the most supported number of ancestral clusters (*K*; denoted to the right of each plot) for empirically informed simulations of genetic drift over time (i.e., generations; denoted to the right of each plot). Simulated populations are labeled by their empirically informed analogs and their effective population sizes are denoted in parentheses. Note that population structure is initially absent at zero generations as populations start with approximately equal allele frequencies (plus or minus sampling error) and populations with lower effective population sizes diverge more quickly. Most populations assign to distinct clusters after 20–30 generations of drift.

## Data Availability

Filtered genotypes and sample metadata have been archived in the Dryad Digital Repository: doi:10.5061/dryad.rbnzs7hk8.
