# Supplementary material for: Complications of Estimating Hatchery Introgression in the Face of Rapid Divergence: A Case Study in Brook Trout (Salvelinus fontinalis)
Source: Evol Appl. 2024 Dec 16;17(12):e70026. doi: 10.1111/eva.70026 (PMC11648973; doi:10.1111/eva.70026)
Supplement: Supplementary file 1 — Data S1. Table S1. Summary of ADMIXTURE population‐specific average assignment probabilities to 14 ancestral clusters. Note that populations largely assign to their own distinct clusters. See Figure 6 for a graphical representation of the results used to calculate these averages. Figure S1. Relationships of genetic diversity and inbreeding coefficients to effective population size for 12 wild brook trout populations and the St. Croix Falls Hatchery strain. Note that the unnamed tributary to the Upper Pine River was excluded from these analyses as its effective population size was unable to be accurately estimated due to recent introgression. Figure S2. Results of empirical ADMIXTURE results using 1–20 ancestral clusters (K). Superscripts denote populations used as wild broodsources (1) or translocation sources (2). Figure S3. ADMIXTURE results using the most supported number of ancestral clusters (K; denoted to the right of each plot) for empirically informed simulations of genetic drift over time (i.e., generations; denoted to the right of each plot). Simulated populations are labeled by their empirically informed analogs and their effective population sizes are denoted in parentheses. Note that population structure is initially absent at zero generations as populations start with approximately equal allele frequencies (plus or minus sampling error) and populations with lower effective population sizes diverge more quickly. Most populations assign to distinct clusters after 20–30 generations of drift. [file EVA-17-e70026-s001.docx]

## Supplementary Material

Table S1. Summary of ADMIXTURE population-specific average assignment probabilities to 14 ancestral clusters. Note that populations largely assign to their own distinct clusters. See Figure 6 for a graphical representation of the results used to calculate these averages.

|  | Cluster | | | | | | | | | | | | | |
| --- | --- | --- | --- | --- | --- | --- | --- | --- | --- | --- | --- | --- | --- | --- |
| Population | 1 | 2 | 3 | 4 | 5 | 6 | 7 | 8 | 9 | 10 | 11 | 12 | 13 | 14 |
| Ash¹ | 0.94 | 0.00 | 0.01 | 0.01 | 0.00 | 0.01 | 0.00 | 0.00 | 0.00 | 0.01 | 0.00 | 0.00 | 0.00 | 0.01 |
| Byrds | 0.00 | 0.97 | 0.00 | 0.00 | 0.00 | 0.00 | 0.00 | 0.00 | 0.00 | 0.02 | 0.00 | 0.00 | 0.00 | 0.01 |
| Fancy | 0.00 | 0.01 | 0.84 | 0.01 | 0.00 | 0.01 | 0.00 | 0.00 | 0.06 | 0.01 | 0.00 | 0.02 | 0.01 | 0.02 |
| Gault Hollow | 0.01 | 0.00 | 0.00 | 0.95 | 0.00 | 0.01 | 0.00 | 0.00 | 0.01 | 0.00 | 0.00 | 0.01 | 0.00 | 0.01 |
| Harker | 0.01 | 0.00 | 0.01 | 0.00 | 0.93 | 0.00 | 0.01 | 0.00 | 0.01 | 0.00 | 0.00 | 0.00 | 0.00 | 0.01 |
| Horse | 0.01 | 0.00 | 0.02 | 0.00 | 0.00 | 0.86 | 0.00 | 0.00 | 0.02 | 0.01 | 0.01 | 0.00 | 0.00 | 0.05 |
| Lawrence² | 0.00 | 0.00 | 0.00 | 0.00 | 0.00 | 0.00 | 0.95 | 0.01 | 0.00 | 0.00 | 0.00 | 0.00 | 0.01 | 0.01 |
| Lowery¹ | 0.00 | 0.00 | 0.00 | 0.00 | 0.00 | 0.00 | 0.06 | 0.90 | 0.00 | 0.00 | 0.00 | 0.01 | 0.00 | 0.01 |
| Marshall | 0.01 | 0.01 | 0.10 | 0.03 | 0.00 | 0.01 | 0.00 | 0.00 | 0.71 | 0.02 | 0.01 | 0.05 | 0.00 | 0.04 |
| Melancthon¹ | 0.00 | 0.01 | 0.00 | 0.00 | 0.00 | 0.00 | 0.00 | 0.00 | 0.00 | 0.97 | 0.00 | 0.00 | 0.01 | 0.00 |
| Upper Pine Trib. | 0.21 | 0.00 | 0.00 | 0.00 | 0.00 | 0.00 | 0.00 | 0.00 | 0.00 | 0.07 | 0.69 | 0.01 | 0.00 | 0.00 |
| St. Croix Falls* | 0.01 | 0.00 | 0.00 | 0.00 | 0.00 | 0.00 | 0.00 | 0.00 | 0.00 | 0.00 | 0.00 | 0.99 | 0.00 | 0.00 |
| South Fork Hay¹ | 0.00 | 0.00 | 0.00 | 0.01 | 0.01 | 0.00 | 0.01 | 0.01 | 0.00 | 0.00 | 0.00 | 0.03 | 0.91 | 0.01 |
| W. Branch Mill¹ | 0.00 | 0.00 | 0.00 | 0.00 | 0.00 | 0.01 | 0.00 | 0.00 | 0.00 | 0.00 | 0.00 | 0.00 | 0.00 | 0.97 |

* Hatchery strain imported from the eastern United States

¹ This population has or is currently being used as a locally derived brood source.

² This population has been used as a wild source for translocations.


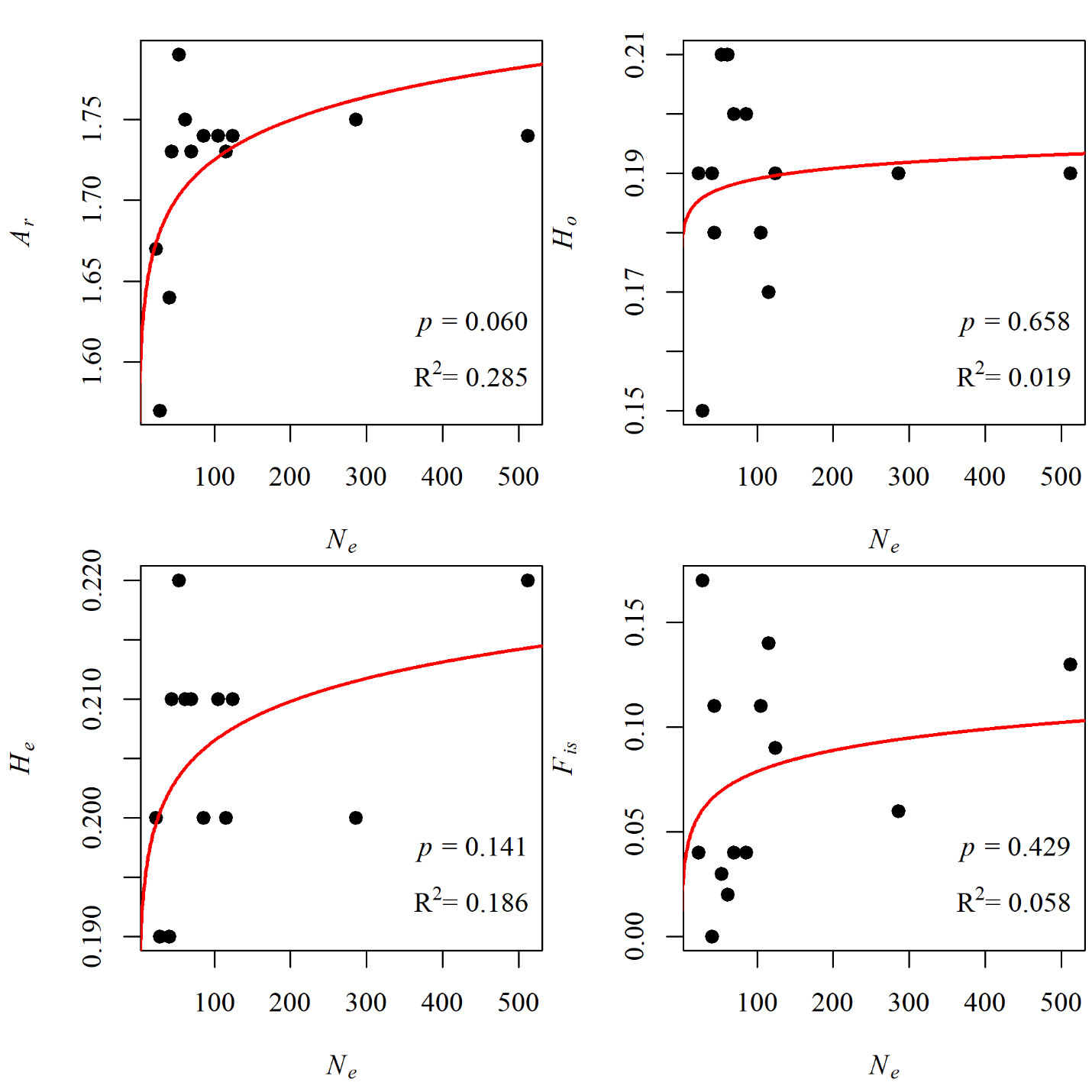


Figure S1. Relationships of genetic diversity and inbreeding coefficients to effective population size for 12 wild brook trout populations and the St. Croix Falls Hatchery strain. Note that the unnamed tributary to the Upper Pine River was excluded from these analyses as its effective population size was unable to be accurately estimated due to recent introgression.


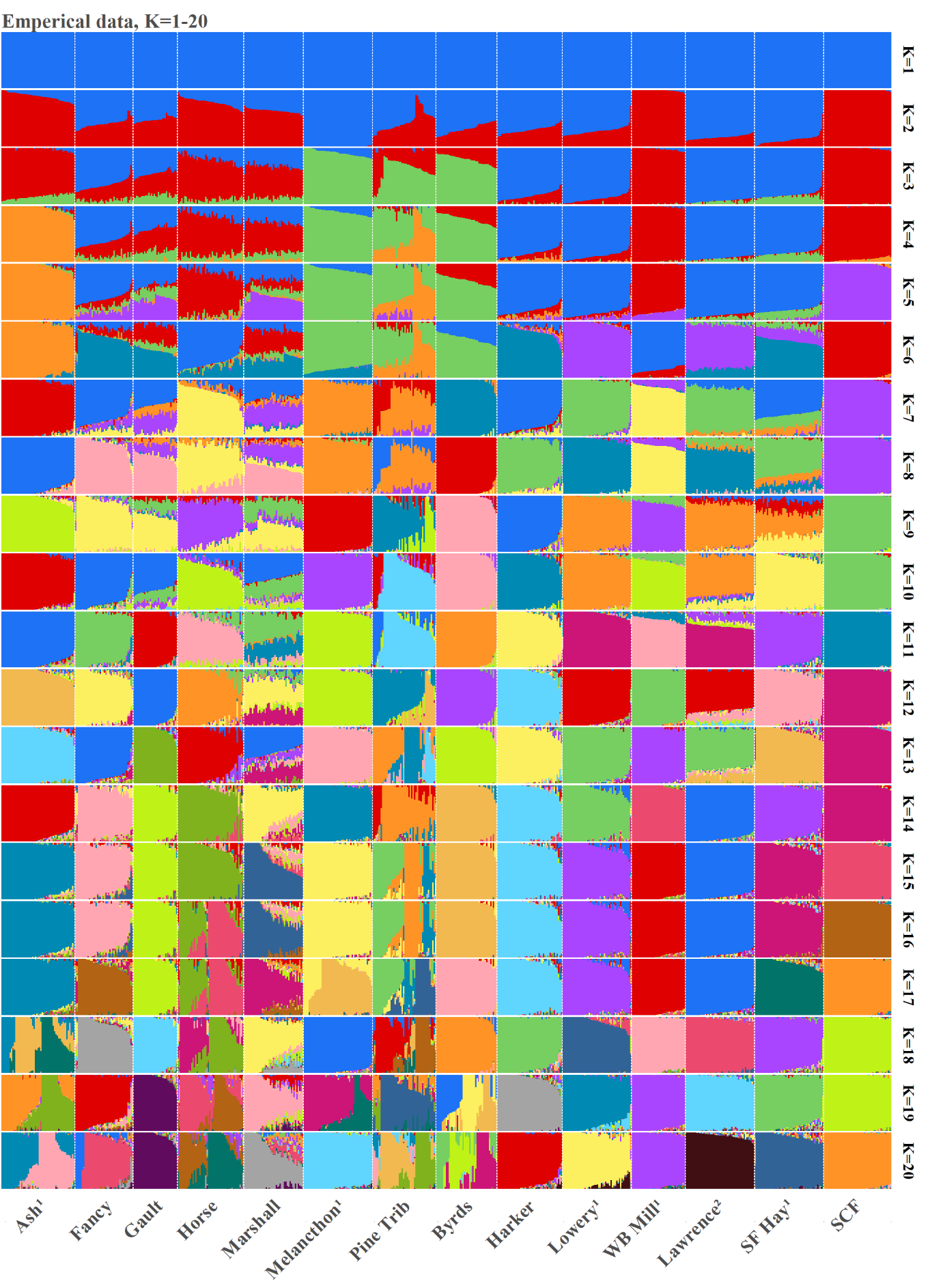
Figure S2. Results of empirical ADMIXTURE results using 1-20 ancestral clusters (*K*). Superscripts denote populations used as wild broodsources (¹) or translocation sources (²).


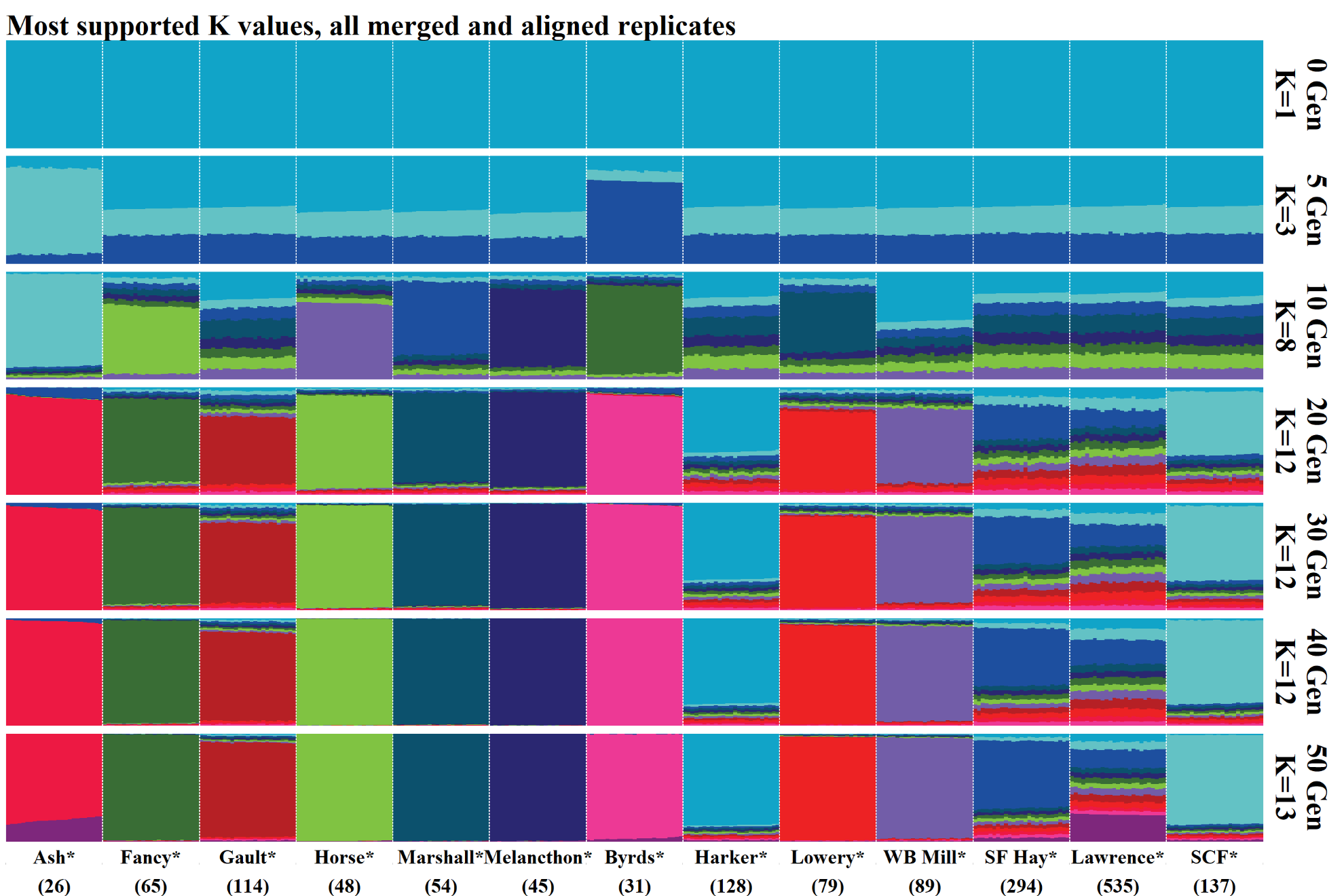


Figure S3. ADMIXTURE results using the most supported number of ancestral clusters (*K*; denoted to the right of each plot) for empirically informed simulations of genetic drift over time (i.e., generations; denoted to the right of each plot). Simulated populations are labeled by their empirically informed analogs and their effective population sizes are denoted in parentheses. Note that population structure is initially absent at zero generations as populations start with approximately equal allele frequencies (plus or minus sampling error) and populations with lower effective population sizes diverge more quickly. Most populations assign to distinct clusters after 20-30 generations of drift.
